# Reducing Ethnic and Geographic Inequities to Optimise New Zealand Stroke Care (REGIONS Care): Protocol for a Nationwide Observational Study

**DOI:** 10.2196/25374

**Published:** 2021-01-12

**Authors:** Annemarei Ranta, Stephanie Thompson, Matire Louise Ngarongoa Harwood, Dominique Ann-Michele Cadilhac, Peter Alan Barber, Alan John Davis, John Henry Gommans, John Newton Fink, Harry Karel McNaughton, Hayley Denison, Marine Corbin, Valery Feigin, Virginia Abernethy, William Levack, Jeroen Douwes, Jacqueline Girvan, Andrew Wilson

**Affiliations:** 1 Department of Medicine University of Otago Wellington New Zealand; 2 Department of Neurology Capital and Coast District Health Board Wellington New Zealand; 3 Department of General Practice and Primary Health Care Faculty of Medical and Health Sciences University of Auckland Auckland New Zealand; 4 Translational Public Health and Evaluation Division, Stroke and Ageing Research in the Department of Medicine School of Clinical Sciences at Monash Health Monash University Melbourne Australia; 5 Faculty of Medical and Health Sciences University of Auckland Auckland New Zealand; 6 Northland District Health Board Whangārei New Zealand; 7 Hawke's Bay District Health Board Hastings New Zealand; 8 Department of Neurology Christchurch Hospital Canterbury District Health Board Christchurch New Zealand; 9 Medical Research Institute of New Zealand Wellington New Zealand; 10 Centre for Public Health Research Massey University Wellington New Zealand; 11 Faculty of Health and Environmental Sciences Auckland University of Technology Auckland New Zealand; 12 Stroke Foundation of New Zealand Wellington New Zealand; 13 Nelson-Malborough District Health Board Neslon-Malborough New Zealand

**Keywords:** stroke, protocols, stroke units, rehabilitation, Māori, Pacific people, health inequities, cost-efficacy, rural, observational study

## Abstract

**Background:**

Stroke systems of care differ between larger urban and smaller rural settings and it is unclear to what extent this may impact on patient outcomes. Ethnicity influences stroke risk factors and care delivery as well as patient outcomes in nonstroke settings. Little is known about the impact of ethnicity on poststroke care, especially in Māori and Pacific populations.

**Objective:**

Our goal is to describe the protocol for the Reducing Ethnic and Geographic Inequities to Optimise New Zealand Stroke Care (REGIONS Care) study.

**Methods:**

This large, nationwide observational study assesses the impact of rurality and ethnicity on best practice stroke care access and outcomes involving all 28 New Zealand hospitals caring for stroke patients, by capturing every stroke patient admitted to hospital during the 2017-2018 study period. In addition, it explores current access barriers through consumer focus groups and consumer, carer, clinician, manager, and policy-maker surveys. It also assesses the economic impact of care provided at different types of hospitals and to patients of different ethnicities and explores the cost-efficacy of individual interventions and care bundles. Finally, it compares manual data collection to routine health administrative data and explores the feasibility of developing outcome models using only administrative data and the cost-efficacy of using additional manually collected registry data. Regarding sample size estimates, in Part 1, Study A, 2400 participants are needed to identify a 10% difference between up to four geographic subgroups at 90% power with an α value of .05 and 10% to 20% loss to follow-up. In Part 1, Study B, a sample of 7645 participants was expected to include an estimated 850 Māori and 419 Pacific patients and to provide over 90% and over 80% power, respectively. Regarding Part 2, 50% of the patient or carer surveys, 40 provider surveys, and 10 focus groups were needed to achieve saturation of themes. The main outcome is the modified Rankin Scale (mRS) score at 3 months. Secondary outcomes include mRS scores; EQ-5D-3L (5-dimension, 3-level EuroQol questionnaire) scores; stroke recurrence; vascular events; death; readmission at 3, 6, and 12 months; cost of care; and themes around access barriers.

**Results:**

The study is underway, with national and institutional ethics approvals in place. A total of 2379 patients have been recruited for Part 1, Study A; 6837 patients have been recruited for Part 1, Study B; 10 focus groups have been conducted and 70 surveys have been completed in Part 2. Data collection has essentially been completed, including follow-up assessment; however, primary and secondary analyses, data linkage, data validation, and health economics analysis are still underway.

**Conclusions:**

The methods of this study may provide the basis for future epidemiological studies that will guide care improvements in other countries and populations.

**International Registered Report Identifier (IRRID):**

DERR1-10.2196/25374

## Introduction

Stroke is the second leading cause of death and disability globally [1]. In New Zealand, the overall burden of stroke is expected to rise by 40% over the next decade, largely due to an aging population [2]. Considerable effort has gone into implementing best practice stroke care across New Zealand in the past two decades; however, substantial variation in stroke service provision continues to exist [3-5].

The low population density of New Zealand has led to concessions in the organization of best practice stroke care being accepted for small- and medium-sized hospitals that serve geographically dispersed urban populations of less than 100,000 people [6]. For example, smaller hospitals are not required to have a designated stroke unit or stroke-specific rehabilitation service, and patients are often managed by clinicians without specific training or regular skill maintenance in stroke care [5]. It is unclear whether equitable patient outcomes can be achieved with these compromises.

Māori, the indigenous population of New Zealand, and Pacific people make up 16.5% and 8.1% of the total population, respectively, and have a greater incidence of modifiable stroke risk factors, including obesity, smoking, hypertension, and diabetes mellitus, compared to other New Zealanders [7,8]. This may explain these populations’ higher age-adjusted incidence of stroke as well as the younger age at first stroke [9], but secondary health service factors that may impact on disparities in stroke outcomes have not been fully explored. Research from other countries shows that ethnic inequities exist in stroke service access and outcomes [10,11], and interpersonal and institutional racism have been shown to exist in general health care in New Zealand [12,13]. Several New Zealand studies have assessed the impact of ethnicity on functional outcomes poststroke, but results are conflicting [14,15]. Therefore, it remains unclear to what extent inequities in access for different ethnic groups may affect patient outcomes beyond differences in baseline risk factors.

Here we describe the design and methods of the *Reducing Ethnic and Geographic Inequities to Optimise New Zealand Stroke Care* (REGIONS Care) study, an investigator-driven, multicenter observational study.

## Methods

### Study Aims

The primary aim of this study is to determine whether there is significant inequity in access to best practice stroke care and patient outcomes in New Zealand, based on the geographic location of health care facilities and the ethnicity of patients. Secondary aims include an exploration of current stroke service access barriers and the impacts of ethnicity, hospital location, and various care pathways on treatment costs and their association with patient outcomes. A further secondary aim is to compare and validate three different stroke data sources to determine the optimal use of health service resources to support data-driven, ongoing service improvement. These data sources include the following: (1) prospective individual patient data collected as part of a formal study involving patient consent for extended patient outcome assessment and data linkage, (2) prospective individual patient data collection as part of a routine national clinical registry that does not involve extended outcome data collection or data linkage and does not require individual patient consent, and (3) use of routinely collected health and other government data involving the New Zealand Integrated Data Infrastructure (IDI) maintained by Stats NZ.

### Primary Hypotheses

We have two primary hypotheses, as follows:

1. Stroke service location and size affects access to optimal stroke care and patient outcomes.
2. Ethnicity affects access to optimal stroke care and patient outcomes.

### Design

**Figure 1:**
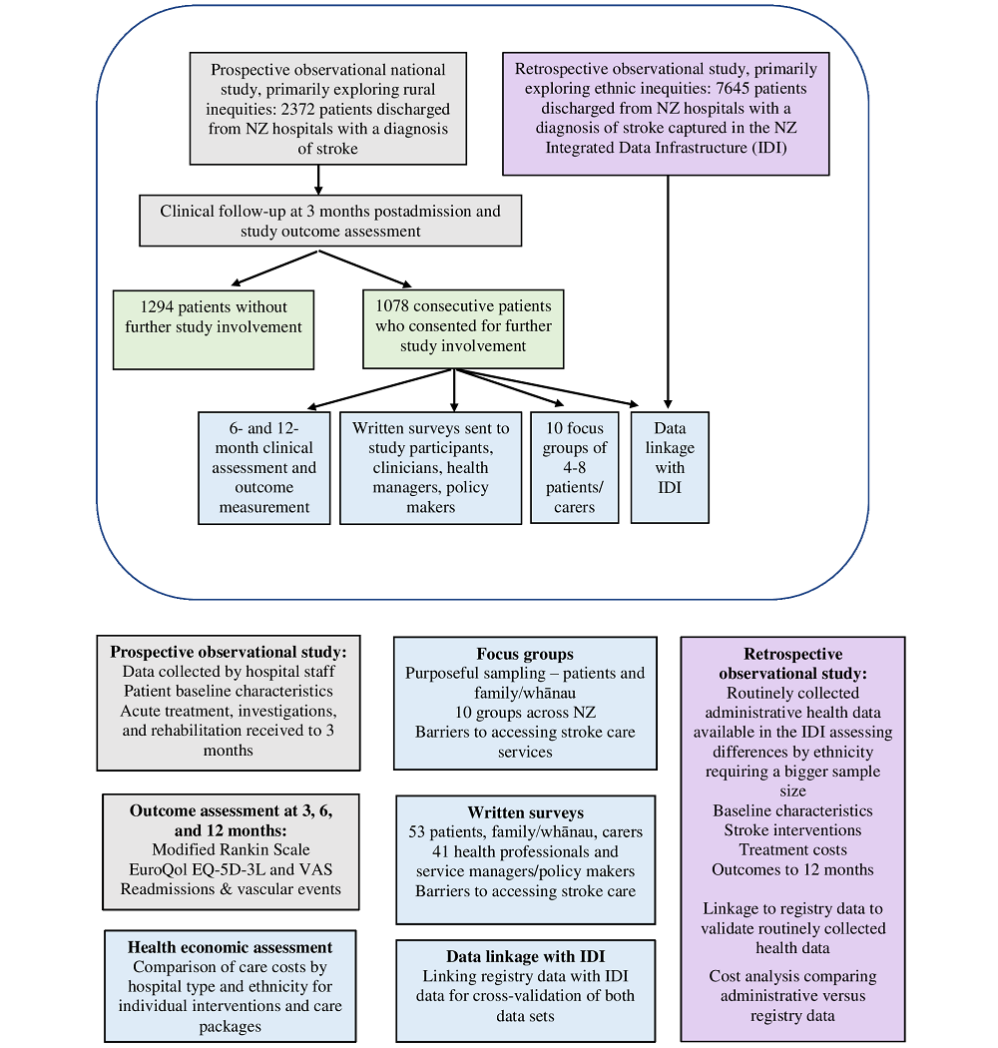
Study flow diagram and components. EQ-5D-3L: 5-dimension, 3-level EuroQol questionnaire; NZ: New Zealand: VAS: Visual Analogue Scale.

This study consists of two large, complementary cohort studies with several nested substudies comprising surveys, focus groups, and an economic evaluation (see Figure 1).

#### Part 1

The two complementary cohort studies are described as follows:

1. Study A is a nationwide, prospective observational study of 2372 consecutively admitted stroke patients cared for at any New Zealand public hospital. The study collects information on individual patient baseline data, to allow for appropriate case-mix adjustments; acute and rehabilitation interventions; treatment costs; and poststroke outcomes up to 12 months. It focuses primarily on differences associated with geographic location of the health service provider.
2. Study B is a nationwide, retrospective observational study of 7645 patients discharged from New Zealand hospitals with a diagnosis of stroke. The study uses routinely collected administrative health data available through Stats NZ’s IDI. The IDI is a longitudinal meta–data set linked at the individual level, consisting of deidentified data from government agencies [16,17]. Baseline characteristics, stroke interventions, treatment costs, and poststroke outcomes up to 12 months will be extracted and validated against a subset of patients recruited as part of Study A from Part 1. This study will focus primarily on differences based on ethnicity for which the greater sample size will provide sufficient power.

#### Part 2

A mixed methods evaluation of access barriers will be carried out. This includes qualitative data derived from 10 focus groups involving face-to-face interviews with selected patients and carers from patients involved in Study A from Part 1; a survey of 50 stroke patients, family members, and/or carers; and a survey of 40 clinicians, health service managers, and/or policy makers.

### Patient Population

For Part 1, Study A, all patients discharged from New Zealand hospitals managing acute stroke between May 1 and July 31, 2018, with a discharge diagnosis of ICD-10-AM (International Statistical Classification of Diseases and Related Health Problems, Tenth Revision, Australian Modification) codes I61, I63, or I64, were captured prospectively into a national stroke registry database. To boost the sample size from smaller hospitals, smaller centers continued data collection until October 31, 2018, or until 100 patients had been recruited, whichever happened first. To boost the tertiary clot-retrieval center sample, tertiary centers collected patients until at least 150 patients had been captured. Nonreperfused patients with complete symptom resolution by 24 hours and no evidence of stroke on imaging (ie, transient ischemic attacks) were excluded. All patients in Part 1, Study A, were invited to consent to further follow-up at 6 and 12 months and data linkage until a preset sample size target (n=1078) was met. Collection of all consecutively admitted patients during the first 3 months, and then until targets are reached, will address the risk of selection bias.

For Part 1, Study B, all patients with the above discharge diagnoses admitted between November 1, 2017, and October 31, 2018, were captured via health administrative data. The only additional inclusion criterion was the requirement for patient consent for study-related follow-up and data linkage.

### Data Collection and Measures

For Part 1, Study A, baseline data include patient demographics, vascular risk factors, premorbid level of function (ie, the modified Rankin Scale [mRS]) [18], employment status, domiciliary information, and level of disability at time of hospital presentation (ie, six simple variable [SSV] model [19]). The mRS is a 6-point scale measuring independence and physical disability, ranging from 0 (no symptoms) to 6 (death) [20]. The SSV model includes six variables: age, living alone prestroke, independence in activities of daily living prestroke, the verbal component of the Glasgow Coma Scale, arm power, and ability to walk on hospital admission. Postadmission data include best practice acute stroke investigations and interventions during acute hospital admission and up to 3 months; in-hospital complications; hospital length of stay; prescription of appropriate secondary prevention medications prior to discharge; if transferred to inpatient rehabilitation, the length of time to transfer and number and duration of therapy contacts during the admission; if referred for community rehabilitation, the time to first assessment and number of therapy contacts in the first 3 months; documentation of interdisciplinary meetings, goal setting, and patient and carer education; provision of culturally appropriate care and support services; and referral to the Stroke Foundation of New Zealand community stroke advisor. The 3-, 6-, and 12-month follow-up assessments captured data from the mRS, the EQ-5D-3L (5-dimension, 3-level EuroQol questionnaire) [21], doctors’ visits, hospital readmissions, work status, and domiciliary status. The complete data collection form can be viewed in Multimedia Appendix 1, Table S1.

For Part 1, Study B, baseline data include patient demographics and risk factors that can be ascertained from available administrative data (see Multimedia Appendix 1, Table S2). Other data include health status at 3, 6, and 12 months poststroke (ie, alive, employed, change in domiciliary address, or deceased) and stroke-related interventions that are captured by administrative data (eg, carotid endarterectomy, endovascular thrombectomy, inpatient rehabilitation, length of hospital stay, and prescriptions filled). A subset of participants from Part 1, Study A, will have their data linked to IDI data to assess the accuracy of the administrative data set.

For Part 2 of the study, health professionals, managers, policy makers, and consented patients and/or their family member or carer were sent an online survey or, where requested, a paper copy. The survey asked participants to rate the accessibility of key stroke interventions and to comment on any barriers to accessing care and potential solutions (see Multimedia Appendix 2 for a sample survey). A subset of patients were also invited to participate in one of 10 focus groups of 4 to 8 people to discuss barriers they have to accessing optimal stroke care and potential solutions. The focus groups were facilitated by experienced research staff and focused on overall experience of stroke care received, description of difficulties in accessing required services, perceived barriers to accessing services, and any suggestions to reducing these barriers. Focus group recruitment was aimed at maximizing diversity in regard to both ethnicity and geographic location.

### Primary Outcome

The primary outcome for Part 1 is *whether there was a* *favorable outcome* at 3 months. For Part 1, Study A, the favorable outcome will be assessed using both an ordinal or *shift* analysis and a dichotomous approach using the mRS. For the dichotomous analysis, a *favorable outcome* is defined as having an mRS score of 0 to 2, and an *unfavorable outcome* is defined as having an mRS score of 3 to 6 [20]. Results will be adjusted for important predictors of outcome, such as stroke severity (ie, SSV model), baseline level of function (ie, mRS), and age. For Part 1, Study B, the mRS is unavailable and a composite measure will be used instead. Here, a favorable outcome is defined as meeting all of the following criteria: being alive; being employed, if employed prestroke; and having no change in domicile location. The latter is intended to capture patients who shifted from home to institutional care or to live with a family member for support, indicating a significant decline in independence. These outcomes are available via the IDI and have been used in previous stroke research [22,23].

### Secondary Outcomes

For both Study A and Study B from Part 1, the mRS at 6 and 12 months comprises the main secondary outcomes. However, best practice stroke care will also be considered as largely based on the Australian Stroke Standards [19]. For Part 1, Study A, this will look at reperfusion therapy, acute stroke unit admission, optimal secondary prevention prescription, transfer to rehabilitation within 7 days of admission, review by a community rehabilitation team member within 7 days of discharge, documented patient-centered goals, documented individualized care plan, and referral to the Stroke Foundation of New Zealand. Other secondary outcomes, which will be measured at 3, 6, and 12 months, where applicable, include the following: hospital readmissions, stroke recurrence, any vascular event or death, discharge destination, treatment costs, quality-of-life assessment using the EQ-5D-3L score, and other interventions received during the hospitalization, such as undergoing relevant imaging, swallow assessment, or a documented continence plan. In addition, the impact of other patient factors on outcome (eg, sex and baseline vascular risk factors) will be assessed. For Part 1, Study B, best practice stroke care measures will include being managed in an acute stroke unit and, if transferred to inpatient rehabilitation, being transferred within 7 days of admission to hospital. These measures align with the New Zealand Ministry of Health quality indicators for optimal stroke processes of care and can be extracted from the National Minimal Dataset, which is a national collection of public and private hospital discharge information, including coded clinical data for inpatients and day patients [24]. Other secondary outcomes that will be obtained from administrative data include the following: the provision of endovascular thrombectomy and the prescription and maintenance of secondary prevention therapies.

### Sample Size Estimates

The main variable of interest is *favorable outcome* as a function of hospital location and ethnicity. Part 1, Study A, of the study is powered on the assumption that favorable stroke outcomes can be achieved among 50% of patients if high-quality care is provided [25]. A sample size of 514 patients per group would identify a discrepancy in favorable outcomes of 10% at 90% power with an α value of .05 between groups. We powered the study to conduct analysis at four geographic levels—tertiary, urban secondary, provincial secondary, and rural secondary hospitals—and aimed to collect data for a minimum of 514 patients from each of these four subgroups. However, because of uncertainty around optimal and suboptimal stroke outcome rates in the New Zealand setting, we chose to make our primary outcome a dichotomous comparison of *urban* versus *nonurban*, and with a sample of 1028 patients per group, the study is powered to allow detection of an intergroup difference of just 7%. To account for a loss to follow-up of 15% to 20%, we aimed for a total sample size of 2400 patients. Due to budgetary constraints, this larger cohort is followed up for 3 months, while a subset of around 1100 patients are followed up for 6 and 12 months, while still meeting the requirements of the primary power calculation assumptions for dichotomous geographic comparisons.

In 2015, there were 944 Māori and 466 Pacific people presenting to hospital with stroke [2,26]; study power will be increased by also capturing all stroke patients admitted to New Zealand hospitals over a 12-month period using the IDI (ie, Part 1, Study B, sample). In 2015, a total of 8495 stroke patients were admitted to hospital with stroke [21]. Allowing for 10% missing or incomplete data, a sample size of 7645 was expected to include 850 Māori and 419 Pacific patients, providing over 90% power to detect a 10% difference in favorable stroke outcomes between Māori and non-Māori patients and over 80% power to detect a difference in favorable stroke outcomes between Pacific and non-Pacific patients.

For the focus groups, the aim is to assemble 10 groups of 4 to 8 participants at locations across New Zealand, covering small, medium, and large district health boards (DHBs) (ie, New Zealand districts). Purposeful sampling according to age (ie, <65 years and ≥65 years), gender, ethnicity (ie, Asian, Māori, Pacific, and European), and place of residence (ie, rural versus urban) was used. The number of planned focus groups is expected to be sufficient to allow for saturation of themes.

### Economic Evaluation

We will describe the costs of stroke in New Zealand using patient-level data. The costs and consequences of those who did and did not receive best practice stroke care based on ethnicity and hospital location will be determined using care pathway analysis methods. Resource use and costs will be obtained as part of the data collection of clinical information for each individual participant. Simulation techniques will be used to assess the identified alternate models of care applied to the New Zealand population. The robustness of results from the incremental cost-effectiveness analyses of each identified care pathway will be assessed using one-way sensitivity analyses and multivariable uncertainty simulations as appropriate for the distribution of the data. Determination of the costs will include the following: index event hospitalization; costs of additional procedures, specifically endovascular thrombectomy; cost of intravenous thrombolysis, if administered; prescriptions; readmissions; aged residential care costs; and lost economic contribution.

### Statistical Analyses

Study A and Study B from Part 1 will examine associations between hospital location, ethnicity, and access to best practice care and stroke outcomes. We will use logistic regression for dichotomous outcome measures and linear regression for continuous outcomes. In Part 1, we will also use ordinal regression to assess associations with the mRS scores. Analyses will be conducted for 3-, 6-, and 12-month posthospital admission, with case-mix adjustments to reduce bias, controlling for important predictors of outcome, including premorbid level of function, level of function at presentation, and age. We will also include baseline characteristics and variables that are associated with the outcome in univariate analyses (*P*<.10) in regression modeling using a backward elimination technique. For Part 1, Study B, stratified analyses will be conducted by age group, ethnicity, domiciliary DHB, treating hospital, premorbid level of function, and domicile.

Regression analyses from Study A and Study B from Part 1 will aid the identification of some potential barriers to accessing best practice stroke care. Barriers will also be assessed in more detail using quantitative and qualitative data from the surveys and focus groups in Part 2. Quantitative survey data will be analyzed using descriptive statistics. Qualitative data from the free-text questions and focus groups will undergo data-driven thematic analysis. Data will be coded to identify thematic patterns. Key themes will be named according to scope and will be defined and described. Results from all data sources will be triangulated to inform conclusions.

### Study Organization, Funding, and Ethics

The study is funded by the Health Research Council of New Zealand (reference 17/037). Ethics approval was received by the Central Health and Disability Ethics Committee (reference 17/CEN/164). The registry-based data collection and 3-month follow-up assessments met New Zealand ethics criteria for *audit*, where data were collected as part of routine care for the purpose of service improvement. In New Zealand, audits do not require individual patient consent. However, the extended follow-ups at 6 and 12 months went beyond routine clinical care and required individual participant consent, which was obtained at the 3-month follow-up assessment. Where a patient was unable to provide consent themselves, consent from someone who carries the responsibility for the participant’s welfare was permitted. Patients approached for consent were also asked for permission to submit their data to Stats NZ for data linkage with other routinely collected government data for validation purposes. Ethics approval included the linkage of patient data to anonymized IDI data for patients who died prior to the 3-month follow-up assessments and who, therefore, were not able to provide consent for data linkage. All data linked via IDI are fully anonymized.

## Results

As of December 2020, data collection was nearly completed. A total of 2379 patients have been recruited for Part 1, Study A; 6837 patients have been recruited for Part 1, Study B; 10 focus groups have been conducted and 70 surveys have been completed in Part 2. Data collection is still underway for Part 1, Study B, and primary and secondary analyses, data linkage, data validation, and health economics analysis are expected to be completed by March 2021.

## Discussion

The study design is significant for several reasons. It involves two concurrent, large observational studies using different but overlapping data sources to ensure sample sizes are achieved in order to answer the main questions while optimizing data quality. The use of Stats NZ’s IDI, an internationally unique and powerful research tool that integrates routinely collected health data and allows linkage to research data, is unprecedented in stroke research. The availability of both data sets within a single study allows for cross-validation, which will provide important information to guide optimal resource use for ongoing and future data collection to continuously drive nationwide stroke service improvement. Involvement of every single stroke hospital in the country is a significant achievement that will provide high-quality epidemiological data and demonstrates that this approach is feasible for future stroke research. Furthermore, the inclusion of patient data from prehospital (eg, arrival mode and detailed premorbid level of function) and following patients through the completion of community rehabilitation adds considerable depth to the data, with the longer-term follow-up of up to 12 months offering important insights into outcomes that are meaningful to consumers. The research team includes academic and nonacademic clinicians from a variety of professional backgrounds (ie, primary, secondary, and tertiary medical care; nursing; therapy; and rural care), epidemiologists, consumers, and stroke support organization representatives. The strong focus on approaching stroke care from an interdisciplinary and overall system perspective, keeping the patient at the center, and recognizing the important contributions that are made throughout the patient’s journey at all of the involved facilities has contributed to our recruitment success and will add valuable insights during the analysis and dissemination phases of the study. Potential limitations include the observational nature of the study, which risks bias, but bias is minimized through the collection of all consecutively admitted patients; the fact that nonhospitalized patients are not included, but fortunately we know that number is very low (~2%) [14]; and the fact that while this is a complete national cohort, this single-nation sample will not be generalizable to all other settings. However, we do expect that findings will be widely informative given many shared resource and ethnic inequity challenges globally.

The results from this study will inform whether changes to New Zealand stroke services are required, with the goal of ensuring that patients receive optimal stroke care and achieve favorable outcomes poststroke, irrespective of ethnicity or geographic location. In addition, the focus groups and surveys will provide information on how to best tackle identified inequities. The health economic analysis will aid DHBs with funding prioritization around proposed service improvements. Finally, the data validation part of the project will provide the New Zealand Ministry of Health with vital information to guide future investments into optimal data-driven, ongoing, stroke service improvement efforts.

## Appendix

Multimedia Appendix 1 Part 1 data dictionary and baseline information from administrative data.

Multimedia Appendix 2 Part 2 patient survey.

Multimedia Appendix 3 Peer review comments from the Health Research Council of New Zealand - Reviewer #97.

Multimedia Appendix 4 Peer review comments from the Health Research Council of New Zealand - Reviewer #47.

Multimedia Appendix 5 Peer review comments from the Health Research Council of New Zealand - Reviewer #45.

## Abbreviations

DHB: district health board
EQ-5D-3L: 5-dimension, 3-level EuroQol questionnaire
ICD-10-AM: International Statistical Classification of Diseases and Related Health Problems, Tenth Revision, Australian Modification
IDI: Integrated Data Infrastructure
mRS: modified Rankin Scale
REGIONS Care: Reducing Ethnic and Geographic Inequities to Optimise New Zealand Stroke Care
SSV: six sample variable
