# Supplementary material for: Reducing Ethnic and Geographic Inequities to Optimise New Zealand Stroke Care (REGIONS Care): Protocol for a Nationwide Observational Study
Source: JMIR Res Protoc. 2021 Jan 12;10(1):e25374. doi: 10.2196/25374 (PMC7838000; doi:10.2196/25374)
Supplement: Multimedia Appendix 1 [file resprot_v10i1e25374_app1.docx]

**Multimedia Appendix 1**

**Table S1. Part 1, Study A, data dictionary**

| Variable/Field name | Definition |
| --- | --- |
| study_ID | Participant study ID |
| study_pt | Is the participant part of HRC study |
| dhb_staff | Was data entry completed by DHB staff – no refers to data entry completed by research staff |
| dhb_present | The DHB where the patient presented to first |
| hosp_present | The hospital where the patient presented to first |
| tertiary | Is the presenting hospital a tertiary centre |
| rural | Is the presenting hospital rural |
| hosp_type | Type of hospital first presented to |
| hosp_transfer1 | The hospital a patient transferred to from the presenting hospital |
| hosp_transfer2 | The hospital a patient transferred to after the first transfer |
| hosp_dc | The hospital from which a patient was discharged from |
| dhb_domicile | The patient’s ‘home’ DHB based on where they live |
| domicile_urban | Is the patient’s home DHB urban or rural |
| region_domicile | The region in which a patient lives |
| nhi | NZ unique national health index number |
| age | Patient age |
| sex | Patient sex |
| ethnicity | The ethnicity with which the patient identifies |
| european | Whether the patient identifies as European |
| maori_pacific | Whether the patient identifies as Māori or one of the Pacific ethnicities |
| pt_identified | The manner in which the patient was identified to add to the database |
| dx_1 | Primary patient diagnosis |
| dx__2 | Secondary patient diagnosis |
| isch_location_1 | Location of ischaemic stroke |
| isch_location_2 | Location of ischaemic stroke |
| isch_cause_1 | Cause of ischaemic stroke |
| isch_cause_2 | Cause of ischaemic stroke |
| ich_location | Location of haemorrhagic stroke |
| ich_cause_1 | Cause of haemorrhagic stroke |
| ich_cause_2 | Cause of haemorrhagic stroke |
| stroke_onset_date | Date stroke symptoms started |
| stroke_onset_time | Time stroke symptoms started |
| onset_date_time | Date/time stroke symptoms started (the above 2 variables combined into one column) |
| wake_up | Whether the patient woke up with stroke symptoms/exact time of symptom onset is unknown |
| known_onset | If the patient woke up with symptoms/exact time unknown, the time that symptoms were first identified |
| onset_time_estimate | An estimate of the time delay between stroke symptom onset and hospital arrival when time and/or date are unknown |
| arrival_mode | The mode of transport the patient took to get to the hospital |
| tx_hosp | The hospital from where a patient was transferred from |
| tx_mode | The type of transport used to transfer a patient between hospitals |
| hospital_arrival | Date and time of hospital arrival |
| Arrival minus onset | Time from onset to arrival (in hours) |
| living_situation | Where the patient was living pre-stroke |
| employment | Whether the patient was in paid employment pre-stroke |
| mrs_pre | Modified Rankin Scale score (level of functional independence) before the stroke |
| svv_gcs | Whether the patient was fully verbal and orientated at time of hospital presentation |
| ssv_walk | Whether the patient could walk without needing help from another person at time of hospital presentation |
| ssv_motor | Whether the patient can lift both arms horizontally at time of hospital presentation |
| ssv_total | Total SSV score – the sum of svv_gcs, ssv_walk and ssv_motor |
| glucose_on_arrival | Blood glucose in ambulance |
| SBP | Systolic blood pressure in ambulance |
| DBP | Diastolic blood pressure in ambulance |
| weight | Patient weight on hospital arrival |
| height | Patient height |
| bmi | Patient body mass index |
| htn1 | Whether the patient has hypertension |
| dm1 | Whether the patient has diabetes |
| lipid1 | Whether the patient has high cholesterol |
| af1 | Whether the patient has atrial fibrillation |
| tob1 | Whether the patient is a smoker |
| stroke1 | Whether the patient has had a previous stroke |
| tia1 | Whether the patient has had a previous TIA |
| ihd | Whether the patient has ischaemic heart disease |
| rhd | Whether the patient has rheumatic heart disease |
| fh | Whether the patient has a family history of stroke |
| cs | Whether the patient has carotid stenosis |
| no_anticoag_reason | The reason the patient is not on anti-coagulation medication for atrial fibrillation |
| cvd_date_mo | Month of the most recent stroke or TIA |
| cvd_date_yr | Year of the most recent stroke or TIA |
| telestroke | Whether Telestroke was used for thrombolysis |
| thrombolysed | Whether the patient was thrombolysed |
| no_tpa | Reason the patient was not thrombolysed |
| dabigatran_reversal | Whether patients on dabigatran received idarucizumab to reverse anticoagulation effect prior to thrombolysis |
| inpt | Whether the patient was in hospital at the time of symptom onset |
| ooh | Whether the patient presented to hospital out of hours (weekends or public holidays or week days 1600hr to 0759hr) |
| needle_time | Time that thrombolysis medication is administered |
| otdt | Time (in minutes) from symptom onset to arrival at hospital |
| dtct | Time (in minutes) from arrival at hospital to having a CT scan |
| cttnt | Time (in minutes) from having CT scan to administration of thrombolysis medication |
| dtn | Time (in minutes) from hospital arrival to administration of thrombolysis medication |
| otnt | Time (in minutes) from symptom onset to administration of thrombolysis medication |
| scr | Whether patient had stroke clot retrieval |
| scr_centre | The centre where patient had stroke clot retrieval |
| vessel_type | Which vessel was occluded |
| scr_arrival | Time of arrival at the clot retrieval centre |
| groin_time | Time of the groin puncture for clot retrieval |
| reperfusion_time | Time the vessel was opened and tissue reperfused |
| tici | Reperfusion score indicating degree of reperfusion achieved |
| post_scr_ct1 | What the CT scan shows after clot retrieval |
| post_scr_ct2 | What the CT scan shows after clot retrieval |
| atgt | Arrival at SCR centre to groin time |
| ntgt | Thrombolysis ‘needle time’ to groin time |
| gtrt | Groin puncture to reperfusion time |
| otgt | Symptom onset to groin time |
| otrt | Symptom onset to reperfusion time |
| complications | Presence and type of complication after reperfusion |
| alive7 | Whether the patient is alive at 7 days after admission to hospital |
| nihss | Stroke scale score (quantifies the impairment cause by stroke) on admission to hospital |
| nihss_post | Stroke scale score at 24-48 hours post admission to hospital |
| asu_admit | Date and time of admission to acute stroke unit |
| hemicraniectomy | Whether the patient had a hemicraniectomy for this event |
| hemicraniectomy_date | Date of hemicraniectomy |
| palliative | Whether the patient received palliative care |
| ct | Whether the patient had a CT scan |
| mri | Whether the patient had a MRI scan |
| cta_cow | Whether the patient had a CTA or MRA of Circle of Willis |
| cta_neck | Whether the patient had imaging of the neck vessels |
| hgbaic | Whether the patient had glycated haemoglobin measured |
| lipids | Whether the patient had lipids tested |
| ecg | Whether the patient had an ECG done |
| tele | Whether the patient had 24 hours or more of telemetry |
| tte | Whether the patient had a transthoracic echo |
| toe | Whether the patient had a transoesphageal echo |
| holter | Whether the patient had 24 hour outpatient Holter monitoring |
| reveal | Whether the patient had Reveal or other outpatient cardiac monitoring |
| brain_scan | Date and time of first brain scan |
| no_scan | Reason no brain scan was completed |
| cus | What carotid imaging shows |
| carotid_endart | Whether patient had carotid endarterectomy |
| carotid_endart_date | Date of carotid endarterectomy |
| telemetry_duration | Number of days of telemetry |
| mobilised | Whether the patient mobilised (out of bed activity) during first 48 hours in hospital |
| asa | Whether the patient had antiplatelet administered within 24 hours (or 48 hours if thrombolysed) |
| cns_date | Date of first stroke CNS assessment |
| cns_time | Time of first stroke CNS assessment |
| no_cns | Reason for no CNS assessment occurred |
| smo | Date of first stroke consultant assessment |
| smo_time | Time of first stroke consultant assessment |
| no_smo | Reason for no stroke consultant assessment |
| swallow_screen | Date of swallow screening assessment |
| swallow_time | Time of swallow screening assessment |
| no_dys | Reason for no swallow screening assessment |
| dysphagia_who | Designation of the person who completed the swallow screening assessment |
| slt_need | Whether a swallow or speech/language problem was identified |
| slt | Date of first SLT assessment |
| slt_time | Time of first SLT assessment |
| no_slt | Reason for no SLT assessment |
| pt | Date of first physio assessment |
| pt_time | Time of first physio assessment |
| pt_48hrs | Number of hours from admission to be seen by physio |
| Pt_48hrs_yesno | Was the patient seen by physio within 48hrs of admission? |
| no_pt | Reason for no physio assessment |
| ot | Date of first OT assessment |
| ot_time | Time of first OT assessment |
| ot_48hrs | Number of hours from admission to be seen by OT |
| ot_48hrs_yesno | Was the patient seen by OT within 48 hours of admission? |
| no_ot | Reason for no OT assessment |
| stroke_nurse | Whether the patient has seen stroke CNS |
| swallow | Whether the patient had a swallow screen |
| physio | Whether the patient has seen physio |
| maori | Whether Māori patient/whanau offered Māori/tikanga support during their care |
| maori_document | If ‘yes’ to Māori/tikanga support, is there documentation of a Māori/tikanga services assessment |
| pacific | Whether Pacific patient/whanau offered Pacific support services during their care |
| pacific_document | If ‘yes’ to Pacific support, is there documentation of a Pacific support services assessment |
| lifestyle_1 | Whether the patient was provided anti-smoking advice (where identified as a smoker) |
| lifestyle_2 | Whether the patient was provided with exercise advice |
| lifestyle_3 | Whether the patient was provided with diet advice |
| lifestyle_4 | Whether the patient was provided with advice if symptoms recur |
| lifestyle_5 | Whether the patient was provided driving advice |
| dvt_proph | Type of DVT prophylaxis in hospital including none |
| complications_1 | Whether the patient had aspiration pneumonia while in hospital |
| complications_2 | Whether the patient had extracranial bleeding |
| complications_3 | Whether the patient had intracranial bleeding |
| complications_4 | Whether the patient had constipation |
| complications_5 | Whether the patient had delirium |
| complications_6 | Whether the patient had depression or anxiety |
| complications_7 | Whether the patient had DVT or PE |
| complications_8 | Whether the patient had fall/s |
| complications_9 | Whether the patient had fever |
| complications_10 | Whether the patient had incontinence |
| complications_11 | Whether the patient had an indwelling catheter |
| complications_12 | Whether the patient had nasogastric feeding |
| complications_13 | Whether the patient had a pressure ulcer |
| complications_14 | Whether the patient had recurrent stroke or stroke progression |
| complications_15 | Whether the patient had seizure |
| complications_16 | Whether the patient had stroke related pain |
| complications_17 | Whether the patient had urinary tract infection |
| complications_18 | Whether the patient had any other complication |
| continence | If incontinence identified, is there a documented continence management plan |
| idc_reason | Reason for the indwelling catheter |
| complication_impact | Whether the complication prolonged the hospital stay |
| asu_dc | Date and time of discharge from acute stroke unit |
| hospital_dc | Date and time of discharge from acute hospital/service |
| drg | Disease related group code used to calculated hospital cost |
| p_holiday_1 | Date of 1^st^ public holiday within audit period (used to calculate working days for therapy intensity) |
| p_holiday_2 | Date of 2^nd^ public holiday within audit period |
| rehab | Whether the patient transferred to inpatient rehab |
| rehab_7days | Number of days to transfer to inpatient rehab from admission |
| pt_ot_attended | The number of physio/OT sessions the patient attended |
| reh_pt_ot_time | Total physio/OT time (in minutes) the patient received during inpatient rehab |
| pt_min/day | Number of minutes/working day of physical therapy patient received (based on LOS and reh_pt_ot_time) |
| bpr_yesno | Did patient achieve transfer to rehab within 7 days and receive 60 mins of physical therapy/working day |
| slt_attended | Number of SLT sessions the patient attended |
| reh_slt_time | Total SLT time (in minutes) the patient received during inpatient rehab |
| slt_min/day | Number of minutes/working day of SLT received during inpatient rehab |
| nutrition_ax | Whether a nutrition assessment was completed and documented |
| dietician | If a nutrition issue identified whether a dietician was involved |
| social_ax | Whether a social needs assessment was completed and documented |
| social_involved | If social needs identified, whether a social worker was involved |
| mood_ax | Whether a mood assessment was completed |
| mood_tool | Tool used to assess mood |
| psych | Where a mood issue identified, whether a psychologist was involved |
| family_mtg | Whether there was a documented family meeting |
| carer | Whether carer training or education was provided prior to discharge |
| home_visit | Whether a home visit was completed prior to discharge |
| rehab_dc | Date of discharge from inpatient rehab |
| rehab_los | LOS in inpatient rehab (in days) |
| dc_med_antiplatelet | Whether the patient was discharged on antiplatelet medication |
| dc_med_statin | Whether the patient was discharged on statin medication |
| dc_med_anti-HTN | Whether the patient was discharged on anti-hypertensive medication |
| dc_med_anticoag | Whether the patient was discharged on anti-coagulation medication |
| gp | Whether the discharge summary was sent to GP with clear follow up plan |
| fu_gp | Follow up to be provided by GP |
| fu_stroke_spec | Follow up to be provided by stroke specialist/neurologist |
| fu_rehab_spec | Follow up to be provided by rehab specialist/geriatrician |
| fu_cns | Follow up to be provided by stroke CNS |
| fu_comm | Follow up to be provided by community team |
| mRS_dc | Modified Rankin Scale score at time of hospital discharge (level of function independence at time of discharge) |
| com_rehab | Whether the patient was referred to the community rehab team |
| time_com | Date of first face to face contact with community rehab team |
| comm_7days | Number of days from hospital discharge till first face to face community rehab contact |
| no_community_date | Reason no date for first community contact is provided |
| type_com | Designation of health professional who saw patient first |
| com_number | Number of community rehab encounters by 3 months post hospital admission |
| com_rehab_post_3_months | Whether community rehab is ongoing beyond 3 months post hospital admission |
| com_dc | Date of discharge from community rehab team |
| sf_ref | Whether the patient was referred to the Stoke Foundation |
| sf_contact | Date of contact with the Stroke Foundation |
| dc_destination | Type of residence the patient was discharged to |
| spec_appt | Number of specialist appointments the patient attended to 3 months post admission |
| readmit | Whether the patient has been readmitted to hospital within 3 months of this stroke event |
| readmit_reason | Reason for readmission |
| alive_3m | Whether the patient is still alive at 3 months following admission |
| date_of_death | Date of death |
| death_cause | Cause of death |
| call_date_3mo | Date of 3 month follow up call |
| dhb_3mo | The DHB where the patient lives |
| call_dhb_staff | Was the call completed by DHB staff – no refers to call completed by research staff |
| health_concerns_3mo | Whether the patient has any ongoing stroke related health concerns at 3 months |
| gp_fu | Whether the patient has seen their GP since they were in hospital |
| gp_number_of_visits_3mo | Number of GP visits |
| spec_fu_3mo | Whether the patient has seen any specialists since they were in hospital |
| spec_num_3mo | Number of specialist visits |
| living_situation_3mo | Where the patient is living at 3 months |
| live_alone_3mo | Whether the patient is living alone at 3 months |
| work_3mo | Whether the hours of work or type of work has changed since/because of the stroke |
| work_change_3mo | Type of change in work status |
| another_stroke_3mo | Whether the patient has had another stroke |
| stroke_num_3mo | Number of additional strokes at 3 months |
| readmit_3mo | Whether the patient has been readmitted to hospital |
| number_readmit_3mo | Number of readmissions at 3 months |
| readmit_reason_3mo_pt | Reason for readmission |
| mrs_3mo | Level of functional independence at 3 months |
| Death_3mo | Was the patient deceased at 3 months? |
| mrs_3mo_dich | Dichotomised mRS score |
| euroquol_mob_3mo | Mobility section of EQ-5D-3L |
| eq_mob_3mo_dich | Dichotomised mobility section of EQ-5D-3L |
| euroquol_cares_3mo | Self care section of EQ-5D-3L |
| eq_cares_3mo_dich | Dichotomised self care section of EQ-5D-3L |
| euroquol_activities_3mo | Usual activities section of EQ-5D-3L |
| eq_activities_dich_3mo | Dichotomised activities section of EQ-5D-3L |
| euroquol_pain_3mo | Pain section of EQ-5D-3L |
| eq_pain_dich_3mo | Dichotomised pain section of EQ-5D-3L |
| euroquol_anxiety_3mo | Depression/anxiety section of EQ-5D-3L |
| eq_anxiety_dich_3mo | Dichotomised depression/anxiety section of EQ-5D-3L |
| euroquol_total_3mo | Health status based on dimensions of mobility, self care, usual activities, pain and anxiety/depression |
| eq_total_dich | Dichotomised EQ-5D-3L total score |
| eq_state_3mo | 5 digit number that describes the patient’s health state |
| eq_index_3mo | Health index score describing patient’s health state |
| health_state_3mo | Self-rated health status score at 3 months |
| call_date_6mo | Date of 6 month call |
| dhb_6mo | The DHB where the patient lives |
| name_6mo | Who conducted the phone call/entered the returned postal questionnaire |
| health_concerns_6mo | Whether the patient has any ongoing stroke related health concerns at 6 months |
| gp_6mo | Whether the patient has seen their GP since they were in hospital |
| gp_num_6mo | Number of GP visits |
| spec_6mo | Whether the patient has seen any specialists since they were in hospital |
| spec_num_6mo | Number of specialist visits |
| living_situation_6mo | Where the patient is living at 6 months |
| live_alone_6mo | Whether the patient is living alone at 6 months |
| work_6mo | Whether the hours of work or type of work has changed since/because of the stroke |
| work_change_6mo | Type of change in work status |
| another_stroke_6mo | Whether the patient has had another stroke |
| stroke_number_6mo | Number of additional strokes at 3 months |
| readmit_6mo | Whether the patient has been readmitted to hospital |
| readmit_number_6mo | Number of readmissions at 6 months |
| readmit_reason_6mo | Reason for readmission |
| mrs_6mo | Level of functional independence at 6 months |
| Death_6mo | Was the patient deceased at 6 months? |
| mrs_6mo_dich | Dichotomised mRS score |
| euroquol_mob_6mo | Mobility section of EQ-5D-3L |
| eq_mob_6mo_dich | Dichotomised mobility section of EQ-5D-3L |
| euroquol_cares_6mo | Self care section of EQ-5D-3L |
| eq_cares_6mo_dich | Dichotomised self care section of EQ-5D-3L |
| euroquol_activities_6mo | Usual activities section of EQ-5D-3L |
| eq_activities_dich_6mo | Dichotomised activities section of EQ-5D-3L |
| euroquol_pain_6mo | Pain section of EQ-5D-3L |
| eq_pain_dich_6mo | Dichotomised pain section of EQ-5D-3L |
| euroquol_anxiety_6mo | Depression/anxiety section of EQ-5D-3L |
| eq_anxiety_dich_6mo | Dichotomised depression/anxiety section of EQ-5D-3L |
| euroquol_total_6mo | Health status based on dimensions of mobility, self care, usual activities, pain and anxiety/depression |
| eq_total_dich_6mo | Dichotomised EQ-5D-3L total score |
| eq_state_6mo | 5 digit number that describes the patient’s health state |
| eq_index_6mo | Health index score describing patient’s health state |
| health_state_6mo | Self-rated health status score at 3 months |
| call_date_12mo | Date of 12 month call |
| dhb_12mo | The DHB where the patient lives |
| name_12mo | Who conducted the phone call/entered returned postal questionnaire |
| health_concerns_12mo | Whether the patient has any ongoing stroke related health concerns at 12 months |
| gp_12mo | Whether the patient has seen their GP since they were in hospital |
| gp_num_12mo | Number of GP visits |
| spec_12mo | Whether the patient has seen any specialists since they were in hospital |
| spec_num_12mo | Number of specialist visits |
| living_situation_12mo | Where the patient is living at 12 months |
| live_alone_12mo | Whether the patient is living alone at 12 months |
| work_12mo | Whether the hours of work or type of work has changed since/because of the stroke |
| work_change_12mo | Type of change in work status |
| another_stroke_12mo | Whether the patient has had another stroke |
| stroke_number_12mo | Number of additional strokes at 12 months |
| readmit_12mo | Whether the patient has been readmitted to hospital |
| readmit_number_12mo | Number of readmissions at 12 months |
| readmit_reason_12mo | Reason for readmission |
| mrs_12mo | Level of functional independence at 12 months |
| Death_12mo | Was the patient deceased at 12 months? |
| mrs_12mo_dich | Dichotomised mRS score |
| euroquol_mob_12mo | Mobility section of EQ-5D-3L |
| eq_mob_12mo_dich | Dichotomised mobility section of EQ-5D-3L |
| euroquol_cares_12mo | Self care section of EQ-5D-3L |
| eq_cares_12mo_dich | Dichotomised self care section of EQ-5D-3L |
| euroquol_activities_12mo | Usual activities section of EQ-5D-3L |
| eq_activities_dich_12mo | Dichotomised activities section of EQ-5D-3L |
| euroquol_pain_12mo | Pain section of EQ-5D-3L |
| eq_pain_dich_12mo | Dichotomised pain section of EQ-5D-3L |
| euroquol_anxiety_12mo | Depression/anxiety section of EQ-5D-3L |
| eq_anxiety_dich_12mo | Dichotomised depression/anxiety section of EQ-5D-3L |
| euroquol_total_12mo | Health status based on dimensions of mobility, self care, usual activities, pain and anxiety/depression |
| eq_total_dich | Dichotomised EQ-5D-3L total score |
| eq_state_3mo | 5 digit number that describes the patient’s health state |
| eq_index_3mo | Health index score describing patient’s health state |
| health_state_12mo | Self-rated health status score at 12 months |
| 1st_recurrent_stroke_date | Date of first recurrent stroke |
| 1st_readmit_date | Date of first readmission |

**Table S2. Baseline information from administrative data**

| **Sex** |
| --- |
| Female |
| Male |
| **Age group** |
| 0-20 |
| 20-39 |
| 49-59 |
| 60-79 |
| 80+ |
| **DHB of Domicile** |
| Northland DHB |
| Waitemata DHB |
| Auckland DHB |
| Counties Manakau DHB |
| Waikato DHB |
| Lakes DHB |
| Bay of Plenty DHB |
| Tairawhiti DHB |
| Hawke's Bay DHB |
| Taranaki DHB |
| Midcentral DHB |
| Whanganui DHB |
| Capital and Coast DHB |
| Hutt Valley DHB |
| Wairarapa DHB |
| Nelson Marlborough DHB |
| West Coast DHB |
| Canterbury DHB |
| South Canterbury DHB |
| Southern DHB |
| *Missing* |
| **Ethnicity** |
| NZ European |
| NZ Māori |
| Pacific Island |
| Asian |
| Other |
| *Missing* |
| **Risk factor: Ever smoked**^£^ |
| Yes |
| No |
| *Missing* |
| **Risk factor: Hypertension** |
| Blood pressure lowering medication*^2^ |
| Hypertension diagnosis in hospital |
| Hypertension composite variable |
| On blood pressure lowering medication at the time of stroke^#^ |
| **Risk factor: Hyperlipidemia** |
| Cholesterol lowering medication*^3^ |
| Hyperlipidemia diagnosis in hospital |
| Hyperlipidemia composite variable |
| On cholesterol lowering medication at the time of stroke^#^ |
| **Risk factor: Diabetes** |
| **Risk factor: Atrial fibrillation** |
| Anticoagulant medication*^1^ |
| Anticoagulant medication* excluding people with pulmonary embolism or DVT diagnoses |
| Anticoagulant medication for more than one year (in 10 years prior to stroke) |
| Atrial firbrillation diagnosis in hospital |
| On anticoagulant medication at the time of stroke^#^ |
| Anticoagulant medication* and prosthetic heart valve diagnosis ever |
| **Risk factor: Prior stroke, TIA, or MI event** |
| **Work status** |
| Paid income taxes the year before stroke |
| **Domicile by meshblock** |
| A meshblock is the smallest geographic unit for which statistical data is collected and processed by Stats NZ. A meshblock can vary in size from part of a city block to a large area of rural land. |
| *Two or more prescriptions in the 10 years prior to stroke event |
| ^#^One or more prescriptions in the 6 months prior to stroke event |
| ^£^This variable is derived from the 2018 Census. Around 16% of the smoking data used to derive this variable has been inputted or taken from 2013 Census data by StatsNZ |
| ^1^Any dispensing of Warfarin sodium, Dabigatran, Rivaroxiban |
| ^2^Any dispensing of Acebutolol, Acebutolol with hydrochlorothiazide, Alprenolol, Amiloride hydrochloride, Amiloride hydrochloride with hydrochlorothiazide, Amlodipine, Atenolol, Atenolol with chlorthalidone, Benazepril, Bendrofluazide, Bendroflumethiazide [Bendrofluazide], Candesartan cilexetil, Captopril, Captopril with hydrochlorothiazide, Carvedilol, Celiprolol, Chlorothiazide, Chlortalidone [Chlorthalidone], Cilazapril, Cilazapril with hydrochlorothiazide, Clonidine, Clonidine hydrochloride, Cyclopenthiazide, Diltiazem hydrochloride, Enalapril maleate, Enalapril maleate with hydrochlorothiazide, Felodipine, Hydralazine hydrochloride, Indapamide, Isradipine, Labetalol, Lisinopril, Lisinopril with hydrochlorothiazide, Losartan, Losartan potassium, Methyclothiazide, Methyldopa, Methyldopa with hydrochlorothiazide, Metoprolol succinate, Metoprolol tartrate, Nadolol, Nifedipine, Oxprenolol, Perindopril, Pindolol, Pindolol with clopamide, Propranolol, Quinapril, Quinapril with hydrochlorothiazide, Sotalol, Timolol, Trandolapril, Triamterene with hydrochlorothiazide, Verapamil Hydrochloride |
| ^3^Any dispensing of Atorvastatin, Ezetimibe, Ezetimibe with simvastatin, Fluvastatin, Pravastatin, Simvastatin |
| ^4^Any dispensing of Aspirin, Clopidogrel |
