# Supplementary material for: Reducing Ethnic and Geographic Inequities to Optimise New Zealand Stroke Care (REGIONS Care): Protocol for a Nationwide Observational Study
Source: JMIR Res Protoc. 2021 Jan 12;10(1):e25374. doi: 10.2196/25374 (PMC7838000; doi:10.2196/25374)
Supplement: Multimedia Appendix 2 [file resprot_v10i1e25374_app2.docx]

**Multimedia Appendix 2: Patient survey**

**Stroke Questionnaire**

Your name (Optional): ____________________________

Which town/region do you live in: ____________________ What is your postcode: _____________

Which best describes you (tick all that apply):

🞏 Person who has experienced a stroke 🞏 Other (please explain):______________________

How would you describe your ethnicity (you may tick multiple boxes): 🞏 NZ European 🞏 Maori 🞏 Pacific Islander 🞏 Asian 🞏 Other: ____________

What is your gender: 🞏 Female 🞏 Male 🞏 Other: ____________

What is your age group? 🞏 <50 🞏 50-70 🞏 70-90 🞏 >90

**Part 1 - Stroke care and access**

1. What service have you experienced after your stroke? Please tick as many as apply.

🞏 Acute Hospital 🞏 Rehabilitation 🞏 Community Stroke Care 🞏 Rest Home

🞏 Other ___________________________

2. How would you rate the stroke care you have received overall?

🞏 Excellent 🞏 Good 🞏 Average 🞏 Below Average 🞏 Poor

Please explain why you chose this rating?:

3. Thinking about specific aspects of stroke care please rate by ticking the appropriate box in each row.

| Stroke care provided by: | Excellent | Good | Below Average | Average | Poor | N/A |
| --- | --- | --- | --- | --- | --- | --- |
| Your GP | 🞏 | 🞏 | 🞏 | 🞏 | 🞏 | 🞏 |
| Ambulance service | 🞏 | 🞏 | 🞏 | 🞏 | 🞏 | 🞏 |
| Emergency department | 🞏 | 🞏 | 🞏 | 🞏 | 🞏 | 🞏 |
| Initial stroke care (First 24 hours) | 🞏 | 🞏 | 🞏 | 🞏 | 🞏 | 🞏 |
| Inpatient ward team | 🞏 | 🞏 | 🞏 | 🞏 | 🞏 | 🞏 |
| Inpatient rehabilitation | 🞏 | 🞏 | 🞏 | 🞏 | 🞏 | 🞏 |
| Community rehabilitation | 🞏 | 🞏 | 🞏 | 🞏 | 🞏 | 🞏 |
| Transitional care facilities | 🞏 | 🞏 | 🞏 | 🞏 | 🞏 | 🞏 |
| Rest home/hospital level care facilities | 🞏 | 🞏 | 🞏 | 🞏 | 🞏 | 🞏 |
| Stroke Foundation support | 🞏 | 🞏 | 🞏 | 🞏 | 🞏 | 🞏 |
| Other, namely: | 🞏 | 🞏 | 🞏 | 🞏 | 🞏 | 🞏 |
|  | 🞏 | 🞏 | 🞏 | 🞏 | 🞏 | 🞏 |
|  | 🞏 | 🞏 | 🞏 | 🞏 | 🞏 | 🞏 |

4. Please tell us about your experience with the stroke care you have received.

What was good?

What needs improvement?

5. Do you have any concerns about how you were treated by health providers during your hospital or post-hospital care? 🞏 Yes* 🞏 No

If Yes please explain:

6. Do you have any concerns that you were unable to access services that you needed during or after your hospital or post-hospital care? 🞏 Yes* 🞏 No

If Yes please explain:

7. Do you have any concerns that some people in New Zealand may be disadvantaged in accessing best stroke care, because of their ethnicity? 🞏 Yes 🞏 No

If Yes please explain:

8. Do you have any concerns that some people in New Zealand may be disadvantaged in accessing best stroke care, because of where they live? 🞏 Yes 🞏 No

If Yes please explain:

9. If you have concerns that some people experience barriers to accessing high quality stroke care please list the barriers that you think exist:

10. Do you have any suggestion of how stroke care access could be improved? 🞏 Yes 🞏 No

If Yes please explain:

11. The government sometimes uses ‘public policy strategies’ (such as ‘tobacco plain packaging’ or a ‘sugar tax’) to help prevent stroke and other chronic conditions. What do you think about this?

12. Smoking, poor diet, and low level of exercise are some known risk factors for stroke. Do you have any suggestions of how exposure to these risk factors may be reduced?

**Part 2 - Centralisation of Care**

Sometimes it is hard for health care teams to know where to best manage a patient. Understandably most people prefer staying closer to their home and family/whanau. However, sometimes more expert care is only available at larger urban hospitals that may be further away. We are interested in learning about your preferences.

13. Do you feel that you received care at the right facility? 🞏 Yes 🞏 No

If NOT were you offered any alternatives? 🞏 Yes 🞏 No

Please explain in more detail:

14. In general, would you prefer staying ‘closer to home’ or being cared for in the ‘most specialised hospital’ in case of a stroke?

🞏 Stay close to home in all circumstances

🞏 Transfer to specialised hospital only if my life is in danger

🞏 Transfer to specialised hospital if they can offer anything that cannot be offered locally

🞏 Transfer to specialised hospital regardless; they have more experience and when I have a stroke I want to get the best possible care to improve the chances of my recovery

🞏 Would depend on other factors

🞏 Not sure

15. What other factors might affect your decision?

16. Which Health Provider would you like to be involved in the decision where to provide your stroke care (tick all that apply):

🞏 I would like the doctor at my local hospital to decide on the right place for me; they will know best

🞏 I would like my GP to be part of this decision

🞏 I would like my local hospital doctor to call a more experienced doctor at the larger hospital to discuss my case to make a decision together

🞏 Other:

17. What level of involvement would you like to have:

🞏 I just want to be told what the doctors think is best and get on with that plan

🞏 I would like to be included in making the decision, but only if my local hospital doctor actively considers transferring me to another hospital

🞏 I would like to be consulted on possible transfer even if my local hospital doctor does not believe I have to transfer somewhere else

18. Do you have any other comments or suggestions?

**This is the end of the Questionnaire**.
