# Supplementary material for: Reducing Ethnic and Geographic Inequities to Optimise New Zealand Stroke Care (REGIONS Care): Protocol for a Nationwide Observational Study
Source: JMIR Res Protoc. 2021 Jan 12;10(1):e25374. doi: 10.2196/25374 (PMC7838000; doi:10.2196/25374)
Supplement: Multimedia Appendix 3 [file resprot_v10i1e25374_app3.pdf]

Applicant peer review report

Health Research Council of New Zealand

Reviewer # 97

Proposal details

Title	Geographic and Ethnic inequities in stroke outcomes
First named investigator	Dr Annemarei Ranta (University of Otago)

Rationale for research Score: 6

The authors clearly lay out the need for such a project, given the burden of stroke, the geographic location, ethnic make up and population density issues that are specific to New Zealand. The rationale is sound.

Design and Methods Score: 5

This study proposes to use existing datasets, plus prospectively-collected data to paint a picture of service delivery for stroke, providing insight into potential differences in stroke service access and outcomes by geographic location and ethnicity.

On first reading of the application the reasons for parts 1a and 1b were not immediately clear. The application would benefit from explicitly stating the research questions that part 1a and part 1b would individually address, what answers would be gleaned from each, and how each part fits into the overall picture of stroke service delivery that is to be generated. The unique contributions of part 1a and part 1b should be highlighted.

I would recommend that a measure of initial stroke severity is included as part of data collection in part 1a (NIHSS). This measure is routinely collected across many existing stroke studies and is a recommendation from the NINDS Common Data Elements Project, ESO and others.

It is unclear what the eligibility criteria for part 1a is. How will consent in those unable to provide consent be dealt with? Will people with post-stroke cognitive impairment (PSCI)/ pre-existing dementia be eligible for inclusion? How will PSCI be recorded? This is a very important factor in examining service needs and efficacy of services, so should be considered.

Which version of the EQ-5D will be used (the 5L version is now available and may increase precision)?

For part 2, it would help to report the questions that each of the focus groups will be asked if possible.

In the sample size calculations section, it would help to know which data the estimates of "50% favourable outcomes despite suboptimal care" are based on, and how a decision of a 10% increase in favourable outcomes was achieved.

On which data are the estimates of data saturation for Part 2 based?

Health significance Score: 6

The disease burden of stroke is well known; the need to optimise service provision to improve outcomes and reduce health inequalities is essential and well argued.

Research Outcomes Score: 6

The research team comprises experts in stroke care, health economics and appropriate stakeholder involvement.

Research Uptake Score: 6

The proposed research appears to make use of an experienced research team, with collaboration from a number of stakeholders who are ready and willing to support implementation of this project. Throughout the application, the practical implications of the research are highlighted, with clear paths to dissemination, sharing of information, implementation strategies and implications for clinical practice and economic impacts.

The application could benefit from including information on data sharing of the raw data from Part 1a. The collection of new information especially in this under-researched population would be of benefit to the wider stroke research community.

General comments

No additional comments to add beyond what has already been said.
