# Supplementary material for: Reducing Ethnic and Geographic Inequities to Optimise New Zealand Stroke Care (REGIONS Care): Protocol for a Nationwide Observational Study
Source: JMIR Res Protoc. 2021 Jan 12;10(1):e25374. doi: 10.2196/25374 (PMC7838000; doi:10.2196/25374)
Supplement: Multimedia Appendix 4 [file resprot_v10i1e25374_app4.pdf]

Applicant peer review report

Reviewer # 47

Proposal details

Title	Geographic and Ethnic inequities in stroke outcomes
First named investigator	Dr Annemarei Ranta (University of Otago)

Rationale for research — Score: 5

A good rationale that is supported by current evidence. Stroke care is not the same throughout NZ. Due to NZs low population density, concessions to best practice care have been accepted for small and medium sized district health boards (DHBs), e.g. smaller hospitals are not required to have a designated geographic stroke unit, or stroke specific rehabilitation service. The authors reviewed previous evidence and have identified gaps in the literature, with a specific focus within New Zealand.

Design and Methods — Score: 4

The aims of the project are to: (1) assess the impact of DHB size and geographic location on stroke service access and patient outcomes; and (2) assess the impact of ethnicity on stroke service access and patient outcomes. The authors report that the study will incorporate a qualitative assessment to assess current access barriers as well as a cost-effectiveness analysis of stroke care in NZ to identify opportunities for improving effectiveness and efficiency. The overall objective is to identify/recommend appropriate models of care to reduce inequities in accessing best practice stroke care to ensure optimal patient outcomes are achieved.

Part A (a) A multi-centre study of 1028 patients recruited via NZ stroke services over 12 months capturing individual patient baseline data (to allow for appropriate case-mix adjustments), interventions, treatment costs, and post-stroke outcome at 3, 6, and 12 months via telephone interviews.

Question: Sample size justified

Part B (b): A larger registry based study following all 8,500 patients discharged from NZ hospitals over 12 months with a diagnosis of stroke for outcomes at 3, 6, and 12 months using administrative health data available through Statistics New Zealand's Integrated Data Infrastructure (IDI).

Question: what is the process of accessing and collating a large database? Do they have the expertise to access the database?

Part 2: A mixed methods evaluation of access barriers using a questionnaire survey of at least 100 patients, 20 clinicians, and 10 policy makers plus qualitative data derived from 8 focus groups involving face-to-face interviews with selected patients and health workers.

Patient Recruitment

Part A (a): All NZ hospital stroke services will be invited to part-take in this study via an existing stroke network mailing list.

Question: what if a DHB does not wish to participate?

Participating centres will be paid a fee for each patient recruited (n=1028) to help off-set the time and resource requirements associated with study participation.

Question: will the study population be representative of Maori and PI people presenting with stroke to NZ hospitals?

Part 1 (b): All NZ patients discharged from NZ public hospitals over a 12 month time frame with a discharge diagnosis of intracerebral haemorrhage, ischaemic stroke/ cerebral infarction, or stroke otherwise unspecified using the ICD-10-AM coding system will be included. Question: all patients – is this realistic?

Authors report allowing for 10% missing/incomplete data a sample of 7645 would be expected to include 850 Māori and 419 Pacific patients providing >90% power to detect a 10% difference in favourable stroke outcomes between Māori and non-Māori and >80% power to detect a difference in favourable stroke outcome between Pacific and non-Pacific people.

Question: what happens if 10% is not achieved? Will the study be under-powered?

Follow-up measurements via telephone interviews at 3, 6 and 12 months with primary and secondary outcomes.

Secondary outcome will be 'best practice stroke care'. Questions: Any evidence supporting for this measure? Any further detail on the cost-effectiveness: i.e. QUALYS being used?

Health significance **Score: 5**

Realistic goals reported by the research team although they state "While contrary to our hypothesis, this is not entirely inconceivable as it has been argued that 'care closer to home' is beneficial to patients and whanau psychologically and that smaller centres have tighter clinician collaboration resulting in fewer challenges around transitions of care." Ministry of Health supports the project. Meets the objectives of HRC criteria.

Research Outcomes **Score: 5**

Good track record with publications and current and previous HRC grant applications. Research team could have reported examples of high impact journals rather than just state international journals. However, at a local and national level the findings will be disseminated.

Research Uptake **Score: 5**

A key strength of the proposal is the direct engagement with consumers and front line stroke clinicians to provide key insights into access barriers which will help facilitate service changes not only at the national and regional, but also the local level.

The results on ethnicity are important in guiding prioritisation of funding for stroke services. Good response with consultation with Māori providers has already begun and has informed study design. The study will not just assess health data, but also directly engage with Māori communities to help identify barriers, and solutions, to current inequities.

General comments

Expertise of team: Wealth of research and clinical experience within stroke. The addition of three members to the team enhances the expertise. (i) A/Prof Dominique Cadilhac is a stroke researcher and health economist from Monash University based in Melbourne; (ii) Ms Jackie Girvan is a current consumer representative on the National Stroke Network; (iii) Ms Ginny Abernethy is the National Stroke Network co-ordinator and works with the Stroke Foundation NZ.
