# Supplementary material for: Reducing Ethnic and Geographic Inequities to Optimise New Zealand Stroke Care (REGIONS Care): Protocol for a Nationwide Observational Study
Source: JMIR Res Protoc. 2021 Jan 12;10(1):e25374. doi: 10.2196/25374 (PMC7838000; doi:10.2196/25374)
Supplement: Multimedia Appendix 5 [file resprot_v10i1e25374_app5.pdf]

Applicant peer review report

Reviewer # 45

Proposal details

Title	Geographic and Ethnic inequities in stroke outcomes
First named investigator	Dr Annemarei Ranta (University of Otago)

Rationale for research Score: 6

The applicants seek to test hypotheses that stroke care (& thus stroke outcomes) varies with location, size of DHB & subject ethnicity. Data will be collected from several sources: new stroke patients will be prospectively recruited and followed serially during recovery; NMDS & IDI databases will be consulted for nationwide access to key stroke interventions and outcomes; qualitative interviews of patients and stroke care providers. The goal of the proposed project is to identify possible inequities in stroke care and recommend correction.

The applicants document ethnic inequalities in stroke outcomes in New Zealand, and outline their plan to determine whether they represent inequities in the delivery of health care. They point out the need to correct for possible effects of disparities in geography, co-morbidities and socioeconomic factors.

Their arguments are presented clearly and succinctly. Their ultimate goal, evidence-informed NZ stroke strategy, should be advanced by this proposed study as planned.

Design and Methods Score: 5

Hypotheses and Aims are stated clearly. Studies have already shown stroke service size and location impacts on access to optimal stroke care and patient stroke outcomes. However, specific geographic peculiarities unique to NZ may be important here. The impact of ethnicity is self-evidently important to study.

The design of the proposed study presents a comprehensive approach to the issue at hand. The prospective portion of Part 1 should provide an accurate snapshot of present stroke service treatment patterns, outcomes and costs. The registry data broadens this to a nationwide perspective. Part 2 is designed to identify potential barriers to optimal care access and quality.

I have a number of concerns about the Methods:

Part 1a:

Although I agree that both ischemic and hemorrhagic stroke patients probably suffer from the same inequalities, including the latter adds a layer of stratification to the analysis that is probably unnecessary. At least in the US, 87% of all strokes are ischemic. Adequate numbers of ischemic strokes should therefore be available to achieve adequate cases for statistical analysis.

The use of both final mRS scores and score change in defining favourable outcome is justified. However, using a simple dichotomy robs the study of considerable statistical power (see below). There may be the need for family consent in cases where the stroke vistim is unable to give informed consent. If no provisions are made, the study risks a bias towards mild stroke.

Part 1b:

I have the same concerns about including hemorrhagic strokes in the mix and in a simple dichotomous primary outcome. I

like the choice of ‘best practice stroke care’ as a secondary outcome.

Part 1 Data Linkage & Analysis:

The applicants admit that the small numbers make it “challenging to recruit a sufficient sample…” This problem will only be exponentially increased for the stratified analyses. More robust approaches, such as sliding dichotomy, ordinal logistic regression, etc. have been shown to add statistical power and reliability to TBI and stroke studies (1,2) using ordinal outcome scales. I strongly suggest the applicants adapt one of these approaches.

I am not an epidemiologist and may not be using the correct terms, but I suspect the applicants rum the risk of ascertainment (sampling) bias when comparing different geographic areas. Surveys such as this tend to find very low or very high frequencies when sampling locales with sparse populations and few observations. The applicants should weight observations of small finite populations or correct estimates of proportions.

Part 2:

No concerns.

Economic evaluation, Sample size calculations:

See Part 1 Data Linkage & Analysis:

References:

1.Murray GD, Barer D, Choi S, Fernandes H, Gregson B, Lees KR, Maas AI, Marmarou A, Mendelow AD, Steyerberg EW, Taylor GS, Teasdale GM, Weir CJ. Design and analysis of phase III trials with ordered outcome scales: the concept of the sliding dichotomy. J Neurotrauma. 2005 May;22(5):511-7.

2.Optimising Analysis of Stroke Trials (OAST) Collaboration., Bath PM, Gray LJ, Collier T, Pocock S, Carpenter J. Can we improve the statistical analysis of stroke trials? Statistical reanalysis of functional outcomes in stroke trials. Stroke. 2007 Jun;38(6):1911-5.

Health significance **Score: 6**

Treatment of stroke has an enormous public health and economic impact. If successful, this study promises to identify impediments to appropriate stroke care in NZ. Evidence discovered from the proposed study can be used to recommend measures to maximize both the quality and the cost-effectiveness of care.

The applicants provide convincing evidence that inequalities exist in NZ in stroke care and outcomes, that the proposed study can address the causes of the inequalities and that correcting those causes is likely to improve outcomes and reduce the cost of care.

Research Outcomes **Score: 7**

Dr Anna Ranta, PI of the proposed project, is young but has spent her career specializing in delivery of stroke care. Recipient of several grants and author of several articles on stroke, she possesses the knowledge, the incentive and the skill to see this project through to successful completion. Her team expresses diversity in representation, both geographically and in terms of expertise. The members are from all areas of New Zealand and include experts both in acute stroke care and rehabilitation, epidemiology, public health, Maori medical issues, health care economics. Also included are a stroke care field worker, 2 study planners and coordinators and a stroke survivor. The proposed time commitment by team members is adequate to achieve the study goals.

Research Uptake Score: 7

The team presents detailed plans to translate evidence obtained from the proposed research project into proposals to update healthcare policy.

General comments
